# Human monocyte-derived suppressive cells (HuMoSC) for cell therapy in giant cell arteritis

**DOI:** 10.3389/fimmu.2023.1137794

**Published:** 2023-02-21

**Authors:** Maxime Samson, Coraline Genet, Marc Corbera-Bellalta, Hélène Greigert, Georgina Espígol-Frigolé, Claire Gérard, Claudie Cladière, Roser Alba-Rovira, Marion Ciudad, Pierre-Henry Gabrielle, Catherine Creuzot-Garcher, Georges Tarris, Laurent Martin, Philippe Saas, Sylvain Audia, Bernard Bonnotte, Maria C. Cid

**Affiliations:** ^1^ Department of Internal Medicine and Clinical Immunology, Dijon University Hospital, Dijon, France; ^2^ Université Bourgogne Franche-Comté, INSERM, Etablissement Français du Sang, Bourgogne Franche-Comté (EFS BFC), UMR1098, RIGHT Interactions Greffon-Hôte-Tumeur/Ingénierie Cellulaire et Génique, Dijon, France; ^3^ Vasculitis Research Unit, Department of Autoimmune Diseases, Hospital Clinic, University of Barcelona, Institut d’Investigacions Biomèdiques August Pi i Sunyer (IDIBAPS), CRB-CELLEX, Barcelona, Spain; ^4^ Department of Ophthalmology, Dijon University Hospital, Dijon, France; ^5^ Department of Pathology, Dijon University Hospital, Dijon, France; ^6^ Centre d'investigation clinique (CIC)-1431, INSERM, Besançon University Hospital, Etablissement Français du Sang (EFS), Besançon, France

**Keywords:** suppressive cells, giant cell arteritis, treatment, cellular therapy, vascular remodeling

## Abstract

**Introduction:**

The pathogenesis of Giant Cell Arteritis (GCA) relies on vascular inflammation and vascular remodeling, the latter being poorly controlled by current treatments.

**Methods:**

This study aimed to evaluate the effect of a novel cell therapy, Human Monocyte-derived Suppressor Cells (HuMoSC), on inflammation and vascular remodeling to improve GCA treatment. Fragments of temporal arteries (TAs) from GCA patients were cultured alone or in the presence of HuMoSCs or their supernatant. After five days, mRNA expression was measured in the TAs and proteins were measured in culture supernatant. The proliferation and migration capacity of vascular smooth muscle cells (VSMCs) were also analyzed with or without HuMoSC supernatant.

**Results:**

Transcripts of genes implicated in vascular inflammation (*CCL2*, *CCR2*, *CXCR3*, *HLADR*), vascular remodeling (*PDGF*, *PDGFR*), angiogenesis (VEGF) and extracellular matrix composition (*COL1A1*, *COL3A1* and *FN1*) were decreased in arteries treated with HuMoSCs or their supernatant. Likewise, concentrations of collagen-1 and VEGF were lower in the supernatants of TAs cultivated with HuMoSCs. In the presence of PDGF, the proliferation and migration of VSMCs were both decreased after treatment with HuMoSC supernatant. Study of the PDGF pathway suggests that HuMoSCs act through inhibition of mTOR activity. Finally, we show that HuMoSCs could be recruited in the arterial wall through the implication of CCR5 and its ligands.

**Conclusion:**

Altogether, our results suggest that HuMoSCs or their supernatant could be useful to decrease vascular in flammation and remodeling in GCA, the latter being an unmet need in GCA treatment.

## Introduction

Giant cell arteritis (GCA) is a large-vessel vasculitis characterized by the association of systemic and vascular inflammation leading to vascular remodeling that triggers the progressive occlusion of affected arteries (1, 2). GCA is usually diagnosed by the identification of lesions of granulomatous vasculitis in a temporal artery biopsy (TAB). TAB also allows researchers to obtain affected tissues in order to better study pathways involved in this vasculitis and ultimately test new treatments (3, 4).

The pathogenesis of GCA is not fully understood but it is accepted that, following activation of dendritic cells of the arterial wall, CD4 T-cells are recruited in the arterial wall and then activate, proliferate and polarize into Th1 and Th17 cells (5). Interferon-gamma (IFN-γ), which is produced by Th1 cells, activates vascular smooth muscle cells (VSMCs) that produce chemokines (CXCL9, -10, 11 and CCL2), triggering the recruitment of monocytes and other CD4 and CD8 T-cells. Mononuclear cells infiltrating the granulomatous lesions in the arterial wall then contribute to producing mediators, such as Platelet-Derived Growth Factor (PDGF) (6) and endothelin-1 (7, 8), triggering the proliferation and migration of VSMCs from the media to the intima, which, together with the production of proteins of the extracellular matrix (collagen-1, collagen-3 and fibronectin), contribute to intimal hyperplasia leading to vascular occlusion and ischemic signs of GCA (5, 9). Therefore, VSMCs play a major role in the pathophysiology of GCA because they facilitate the recruitment of inflammatory cells and also produce extracellular matrix components that may contribute to stenosis of the vascular lumen and generate the ischemic signs of GCA. Previous data have demonstrated that the migration and proliferation of VSMCs, which support this remodeling process, is mainly due to an excess of endothelin-1 and PDGF in GCA lesions (6–8, 10). In addition, a neoangiogenesis process due to increased VEGF production contributes to increased inflammation and vascular remodeling (5).

Glucocorticoids (GCs) are the cornerstone of GCA treatment. They are very effective but must be given for more than one year to avoid relapses, meaning that the great majority of GCA patients develop GC-related complications, which cause significant morbidity and disability (11). In addition, GCs have no significant effect on vascular remodeling processes, which might be involved in the occurrence of relapses and cardiovascular events during the follow-up of these patients (3). To date, only methotrexate and tocilizumab have been shown to be effective for the treatment of GCA, especially to decrease the frequency of relapses and spare GCs (12, 13). Other molecules are being developed but most target inflammation and very few vascular remodeling, which remains an unmet need for improving the outcome of GCA patients (14).

Immunosuppressive/regulatory immune cell-based therapy is a relatively recent approach that may make it possible to reduce doses of immunosuppressive drugs and GCs in the treatment of autoimmune diseases. Along this line, our team has recently reported on the development of a novel clinically relevant and feasible approach to generate *ex vivo* human suppressor cells of monocytic origin (CD33^+^CD11b^+^CD14^+^CD163^+^CD206^+^HLA-DR^+^ cells), referred to as “human monocyte-derived suppressive cells” (HuMoSCs) (15). HuMoSCs are highly potent at inhibiting the proliferation and activation of autologous and allogeneic effector T-cells *in vitro* and *in vivo* (15). Interestingly, HuMoSCs express CCR5 (15), the receptor of CCL3, CCL4 and CCL5, making them theoretically able to home to inflammatory lesions in order to exert their inhibitory functions.

*Ex vivo* generated autologous HuMoSCs or their supernatant could therefore represent a new therapeutic tool to prevent inflammation and vascular remodeling and thus improve the prognosis of GCA patients, alone or in combination with other immunosuppressive drugs. To address this question, we used an *ex vivo* temporal artery culture model to investigate the effect of HuMoSCs on the main pathways of inflammation and vascular remodeling. We then used cultures of VSMCs to study the effect of HuMoSCs on VSMC migration and proliferation, two functions of these cells that play a major role in vascular remodeling (5).

## Materials and methods

### Patients

Fresh TABs with histopathological features of GCA (TAB+) were obtained from twelve newly diagnosed GCA patients. Patients were prospectively enrolled at the Vasculitis Research Unit, Department of Systemic Autoimmune Diseases, Hospital Clinic, University of Barcelona (Spain) and the Department of Internal Medicine and Clinical Immunology, Dijon University Hospital, Dijon (France) after obtaining their written informed consent in accordance with the Declaration of Helsinki (Collection DC-2016-2675). Usual clinical, biological and therapeutic data were recorded at diagnosis. The patients’ main clinical and biological characteristics are summarized in **Supplementary Table 1**.

In addition, 9 fresh TABs without features of GCA (negative TABs) were obtained. Among these 9 patients, none had GCA, 4 had polymyalgia rheumatica (PMR) without associated GCA and the other 5 had an inflammatory syndrome unrelated to GCA, PMR or any other vasculitis.

### HuMoSC generation and preparation of HuMoSC supernatant

HuMoSCs were generated *ex vivo* from circulating monocytes, as already described (15). Briefly, peripheral blood mononuclear cells (PBMCs) were obtained by Ficoll density gradient centrifugation. Then, monocytes were purified by Percoll density gradient centrifugation and cultured in X-Vivo medium (Lonza^®^) supplemented with GM-CSF (10 ng/mL) and IL-6 (10 ng/mL) (Miltenyi Biotec^®^). Cultured cells were harvested after seven days by gentle scraping. At this stage, the cells could be frozen in liquid nitrogen, perhaps transported and thawed within three months. To obtain HuMoSCs, a CD33 positive selection was finally performed according to the Miltenyi kit and protocol for human CD33 isolation (AutoMACS Pro, Miltenyi Biotec^®^). The purity of CD33^+^ isolation was checked by flow cytometry (anti-CD33 FITC, clone HIM 3-4, eBioscience) and was always >80%. Otherwise, cells were not kept for subsequent experiments. With this method, even if generated in the presence of IL-6 and GM-CSF, which usually have a proinflammatory effect, these cells have strong immunosuppressive properties *in vitro* and in an *in vivo* mouse model of Graft-versus-Host-Disease (15).

For preparation of HuMoSC supernatant, HuMoSCs were cultured (10^6^ cells/mL) in complete RPMI for 48 hours before collection.

### Ex vivo cultures of temporal artery

Sections of TAB specimens affected by GCA were embedded in MATRIGEL**^®^** (Dominique Dutscher SAS, Belgium) and cultured as previously described (3). Briefly, surrounding tissue was carefully removed to keep the temporal artery before regular sections of ~1 mm in thickness were cut. These sections were embedded in 25 µL of MATRIGEL^®^ in a 24-well plate (1 section/well and 2 sections/condition) and then covered by 750 µL of RPMI 1640 medium supplemented with 10% fetal bovine serum, 2 mM L-glutamine, amphotericin B at 2.5 μg/ml and gentamycin at 200 μg/ml. On day 1, HuMoSCs (250.10^3^ cells/mL) or HuMoSC supernatant (25%) were added. After five days of culture, arterial sections were collected and homogenized in TRIzol reagent using a Minilys homogenizer (Bertin instruments^®^) before extraction of total mRNA.

### RT-PCR

Total RNA was extracted from cultured artery and cDNA obtained by reverse transcription using a random hexamer priming kit (Thermo Fisher Scientific, Applied Bioscience) in a final volume of 100 µL. Then, specific pre-developed TaqMan probes from Thermo Fisher Scientific (TaqMan Gene Expression Assays) were used for PCR amplification (listed in **Supplementary Table 2**). Fluorescence was detected with the CFX96TM Real-Time PCR Detection System and the results analyzed with the CFX ManagerTM software (Bio-Rad Laboratories). Gene expression was normalized to the expression of the endogenous control *GUSB* using the comparative ΔCt method. mRNA concentration was expressed in relative units with respect to *GUSB* expression (relative expression).

### Cytokine assays

PDGF-AA, PDGF-BB, fibronectin, COL1α1 and VEGF concentrations were measured in the supernatant of *ex vivo* TAB cultures by Luminex^®^ (R&D Systems, Bio-Techne) following manufacturer instructions. Data were acquired and analyzed on a Bio-Plex^®^ 200 system (Bio-Rad, France).

### Generation of vascular smooth muscle cells (VSMCs)

VSMCs were isolated from negative TABs. Briefly, healthy arteries were embedded in MATRIGEL^®^ to ensure prolonged survival and then covered by DMEM supplemented with 10% fetal bovine serum, 2 mM L-glutamine, amphotericin B at 2.5 μg/ml and gentamycin at 200 μg/ml. Cells were treated with trypsin-EDTA (Gibco) between each passage. VSMCs were used after 4–7 doubling passages.

### Scratch-wound healing tests

VSMCs were cultured in 6-well plates with or without human PDGF-AB (R&D Systems, 20 ng/mL) to stimulate VSMC migration [6], and HuMoSC supernatant at 10% or 25%. After 72 hours, the medium was removed and replaced by fresh medium with the corresponding additives and molecules. VSMCs reached confluence after six days of culture. One scratch per well was done using a sterile 10 µL pipette tip. Three pictures per well at different levels were automatically taken using phase-contrast microscopy (Axio Observer 7, Zeiss) every 6 hours for 48 hours. The videos were analyzed by the CellImaP Platform (University of Burgundy, Dijon, France).

### Proliferation assay for VSMCs

VSMCs were plated in flat-bottom 96-well plates at 4,000 cells/well in triplicate wells and incubated at 37°C in 5% CO_2_ for 1 to 7 days. VSMCs were cultured with fresh DMEM (10% FBS), with or without human PDGF-AB (R&D Systems, 20 ng/mL) to stimulate VSMC proliferation [6], HuMoSC supernatant at 10% or 25% and Dexamethasone at 0.5 μg/ml. After 2, 4 and 7 days of culture, cells were fixed and stained with 0.2% crystal violet (Sigma-Aldrich) in 20% methanol for 20 minutes. Wells were washed, air-dried and solubilized in 1% sodium dodecyl sulfate (SDS). Optical density was measured with a plate reader (Multiskan Ascent, Thermo Scientific) at 620 nm wavelength to assess the proliferation of VSMCs.

### Western blot

Cell lysates were obtained using modified radioimmunoprecipitation assay (RIPA) buffer supplemented with protease inhibitors (Complete, Boehringer Mannheim, Mannheim, Germany), NaF (50 mM), PMSF (1 mM), aprotinin, leupeptin, pepstatin (1 ng/ml) and orthovanadate (2 mM) (Sigma-Aldrich). The following primary antibodies were used for immunodetection: rabbit anti-total AKT, anti-phospho-AKT (Thr 308) and anti-phospho AKT (Ser 473) and used at 1:1000 dilution (cell signaling), rabbit anti-total Erk1/2 (dilution 1:2000) and rabbit anti-phospho-Erk1/2 (Thr202/Tyr204) (dilution 1:1000) (cell signaling), mouse anti β-actin used at 1:5000 dilution (Sigma-Aldrich) and rabbit anti-vinculin used at 1:30000 dilution (Abcam). Goat anti-rabbit IgG HRP-linked (cell signaling) and goat anti-mouse IgG HRP-conjugate (Bio-Rad) were used as secondary antibodies at 1:2000 dilution.

### mTOR pathway analysis by confocal microscopy

Cultured artery sections were fixed in cold paraformaldehyde (4%), followed by exposure to increasing concentrations of saccharose (15–30%). Sections were then embedded in optimal cutting temperature OCT (Sakura, Flemingweg, The Netherlands) and stored at -80°C. Then, 10 μm cryostat sections were fixed, blocked and incubated with primary antibodies against human phospho-S6 ribosomal protein (p-rpS6, Ser 235/236, Cell Signaling, at 1:200 dilution) and α-smooth muscle actin (polyclonal, Abcam, at 1:500 dilution), and then donkey secondary antibodies were conjugated with fluorochromes A488 (green) and A647 (red) (Abcam, at 1:300 dilution). We used DAPI to stain nuclei (blue). Visualization was performed with a Leica TCS SP8 fluorescence microscope available at the DimaCell Platform (INRAE, University of Burgundy, Dijon, France).

### Flow cytometry

Membrane labeling was performed on HuMoSCs. Antibodies were incubated for 30 minutes at 4°C in PBS with 0.5% FBS. Antibodies are listed in **Supplementary Table 3**. Data were acquired on a BD Bioscience Canto II cytometer and analyzed with FlowJo^®^ v10.

### Chemotaxis assay

HuMoSC chemotaxis was assessed using Boyden chambers with 10 μm pore polycarbonate filters. The lower chamber was filled with undiluted supernatants of control TAB or GCA-TAB cultivated for five days in MATRIGEL. A total of 30.10^3^ HuMoSC/well in 50 µL of 10% FBS RPMI medium were loaded in the upper chamber. In selected chambers, an antagonist of CCR2 (Calbiochem, used at 100 nM) or CCR5 (maraviroc, R&D Systems, used at 1 µM) was added. After 4.5-hour incubation, the polycarbonate membrane was stained with hematoxylin and eosin and washed before counting number of cells/field.

### Statistics

Data are expressed as numbers (%) for categorical variables and median (IQR) or mean (SEM) for continuous variables. Mann–Whitney or paired Wilcoxon tests were performed to compare continuous variables, as appropriate. Statistical significance was set at P<0.05 (two-tailed). GraphPad Prism^®^ was used for statistical analyses.

## Results

### HuMoSCs reduce markers of vascular inflammation

**Figure 1:**
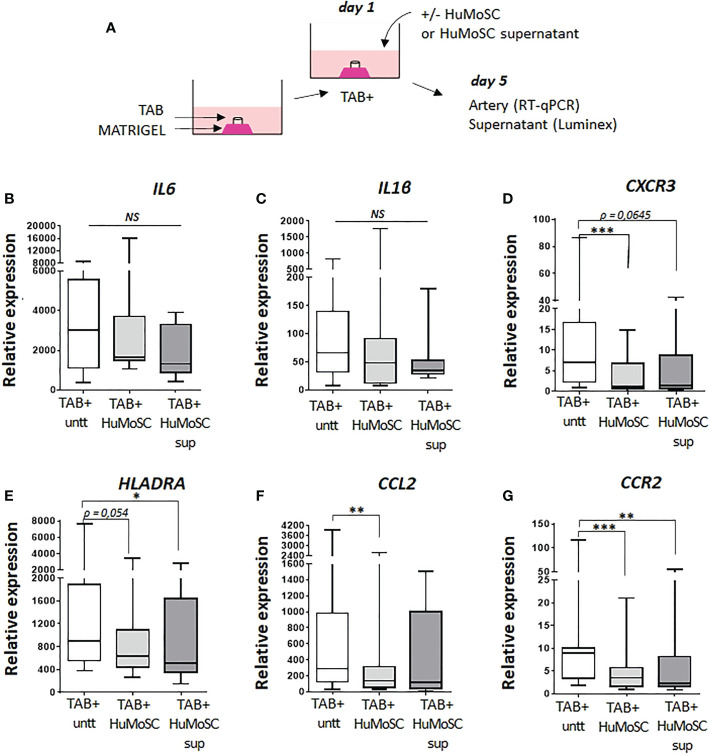
Effect of HuMoSC on vascular inflammation. **(A)** representative method for culture of temporal arteries (TABs): sections of 1 mm of fresh TABs with lesions of GCA (TAB+) were embedded in MATRIGEL. At day 1, HuMoSC (250.10^3^ cells/mL) or 25% of HuMoSC supernatant was added. Each condition was performed in duplicate. After 5 days of culture, arterial sections were collected to measure mRNA by RT-PCR and the supernatant was used to measure proteins. **(B-G)** mRNA expression of genes implicated in vascular inflammation. Experimental conditions: untreated TAB+ (n=12), TAB+ treated with HuMoSC (n=11) or of HuMoSC supernatant (n=10). Whisker boxes show the median (horizontal bar), the IQR (limits of the box) and extreme values (error bars). *P* is the result of Wilcoxon tests. Significant results are shown (*P < 0.05, **P < 0.01 and ***P < 0.001, NS, Not Significant) otherwise non-significant.

**Figure 1** depicts analysis of the effect of HuMoSCs or their supernatant in a model of *ex vivo* cultures of temporal arteries (**Figure 1A**). The mRNA expression of genes of the main markers of vascular inflammation in cultured arteries is reported in **Figures 1B–G**: proinflammatory cytokines (*IL1B* and *IL6*), markers of proinflammatory monocytes/macrophages and Th1 cells (*CCL2* and *CCR2*), markers of Th1 cells (*CXCR3)* and markers of activated antigen-presenting cells (*HLADRA*). In the presence of HuMoSCs, we observed a significant decrease in the level of expression of *CXCR3*, *CCL2* and *CCR2* genes and a tendency for a decrease in *HLADRA* (P=0.054). HuMoSC supernatant had a relatively similar effect with a tendency to be less powerful than HuMoSCs.

### HuMoSCs reduce markers of arterial remodeling

**Figure 2:**
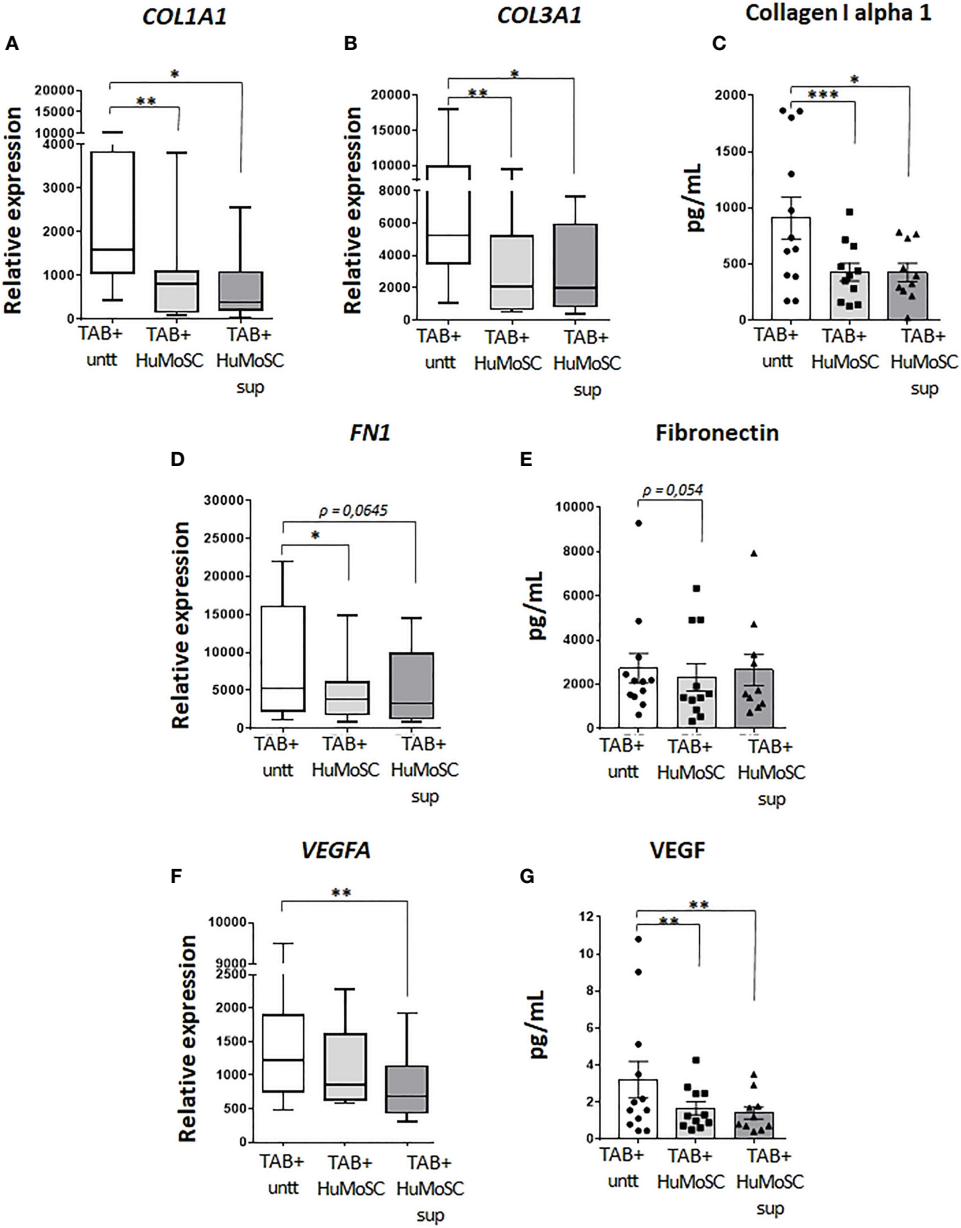
Effect of HuMoSC on vascular remodelling. Sections of fresh temporal arteries (TABs) affected by GCA (TAB+) were embedded in MATRIGEL. At day 1, HuMoSC (250.10^3^ cells/mL) or 25% of HuMoSC supernatant was added. Each condition was performed in duplicate. After 5 days of culture, arterial sections were collected to measure mRNA by RT-PCR and the supernatant was used to measure proteins. **(A-E)** effect of HuMoSC on the production of matrix proteins: mRNA expression in TAB+ **(A, B, D)** and protein level in the supernatant **(C, E)**. **(F, G)** effect of HuMoSC on angiogenesis: mRNA expression of *VEGFA* in TAB+ **(F)** and concentration of VEGF in the supernatant of culture **(G)**. Experimental conditions: untreated TAB+ (n=12), TAB+ treated with HuMoSC (n=11) or of HuMoSC supernatant (n=10). Whisker boxes show the median (horizontal bar), the IQR (limits of the box) and extreme values (error bars). Histograms show the median (horizontal bar) and the IQR (error bars). *P* is the result of Wilcoxon tests. Significant results are shown (*P < 0.05, **P < 0.01 and ***P < 0.001) otherwise non-significant.

Proteins that compose the extracellular matrix were also assessed in the model of *ex vivo* cultured arteries. The effect of HuMoSCs or their supernatant was similar even if the latter was less powerful: dramatic decrease in the mRNA and protein levels of the alpha-1 chain of collagen-1 (**Figures 2A, C**), significant decrease in the mRNA level of *COL3A1* (**Figure 2B**) and a tendency to a decrease in the mRNA and protein levels of fibronectin (**Figures 2D, E**).

Angiogenic factors were assessed by measuring the mRNA level of *VEGFA* and the concentration of VEGF in the supernatant of *ex vivo* cultures of arteries. By contrast with HuMoSC supernatant, HuMoSC cells had no impact on the mRNA level of VEGFA (**Figure 2F**). However, both HuMoSC cells and supernatant induce a dramatic decrease in the secretion of VEGF (**Figure 2G**), suggesting an anti-angiogenic effect of HuMoSCs.

Taken together, these results suggest an ability of HuMoSCs to inhibit vascular remodeling, prompting us to investigate this therapeutic potential in a single-cell model using VSMC cultures as these cells are the main actor of vascular remodeling in GCA (5, 9, 16). To retain a single model and avoid any allogeneic stimulations, further experiments were performed in the presence of HuMoSC supernatant rather than HuMoSCs.

### HuMoSCs decrease the migration and proliferation of VSMCs

**Figure 3:**
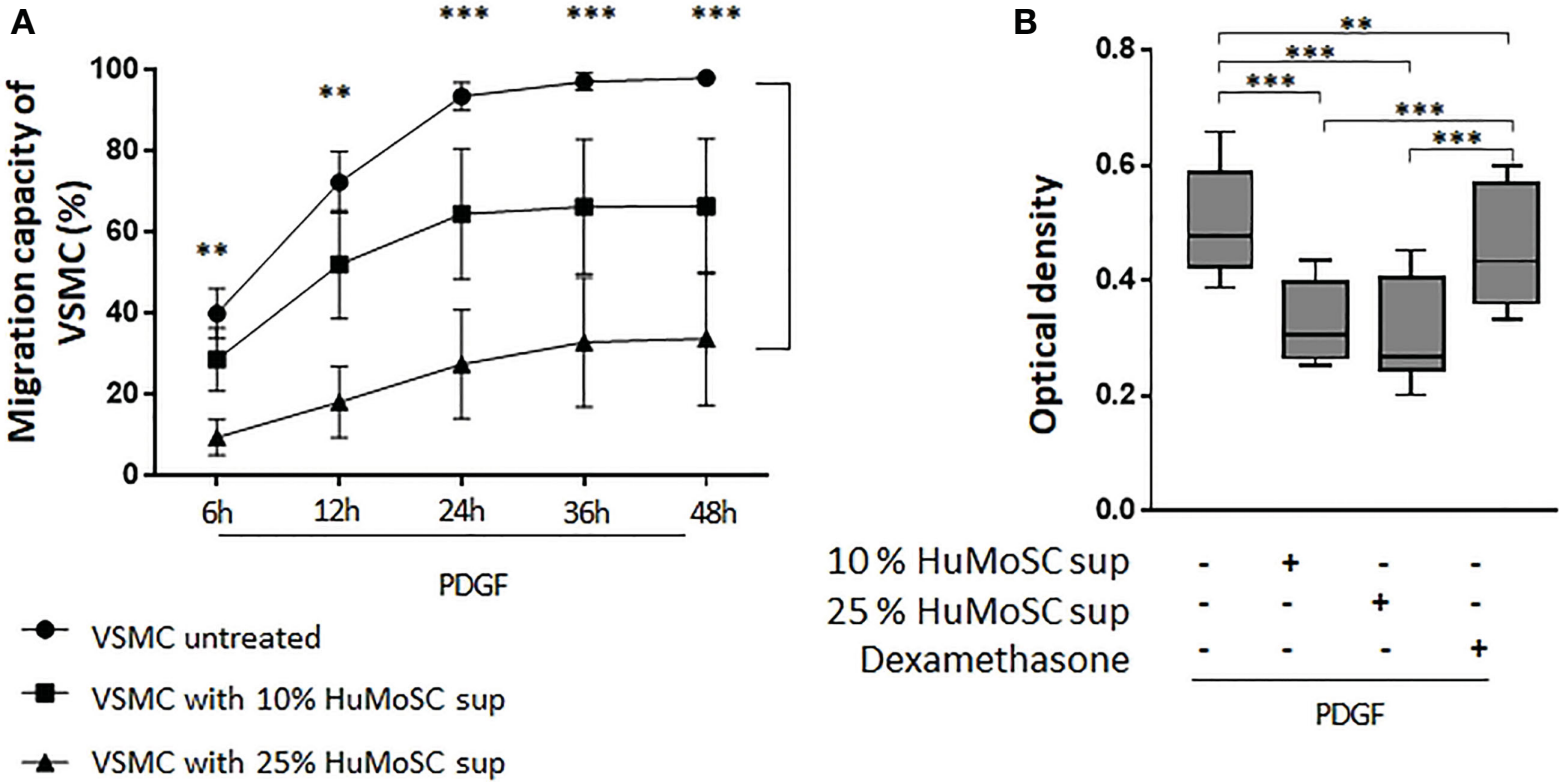
Effect of HuMoSC on migration and proliferation of VSMC. **(A)** Study of the migration of VSMC in the presence of PDGF-AB (20 ng/mL). Scratch assays were used to measure VSMC migration capacity. Time-lapse video microscopy recorded images during 48h. HuMoSC supernatant treatment was used at 10% or 25% in VSMC cultures. Data are presented as mean percentage of wound healing ± SEM (n=3 independent experiments). **(B)** Proliferation of VSMC was measured by optical density after treatment with crystal violet after 7 days of culture. VSMC were cultured with PDGF (20 ng/ml), dexamethasone (0,5 μg/ml) and/or 10% or 25% of supernatant of HuMoSC. Whisker boxes show the median (horizontal bar), the IQR (limits of the box) and extreme values (error bars). Results show triplicates from 4 independent experiments. *P* is the result of Mann Whitney tests **(A)** or Wilcoxon tests **(B)**. **P < 0.01, ***P < 0.001.

PDGF plays an important role in the activation of vascular remodeling related to VSMC proliferation and migration in GCA (6). We therefore used this factor to stimulate VSMC migration and proliferation in *in vitro* experiments. We clearly demonstrated that HuMoSC supernatant inhibits in a dose-dependent manner the migration of VSMCs previously activated with PDGF-AB. Even after 48 hours of culture, a treatment with HuMoSC supernatant kept the scratch open (**Figure 3A**).

Moreover, HuMoSC supernatant also induces dose-dependent inhibition of the proliferation of VSMCs previously activated with PDGF-AB (**Figure 3B**). Interestingly, the inhibitory effect of HuMoSC supernatant was much stronger than that of dexamethasone.

Taken together, these results suggest that HuMoSC supernatant have an anti-vascular remodeling effect through its ability to interfere with the PDGF pathway.

### HuMoSCs decrease PDGF receptors

**Figure 4:**
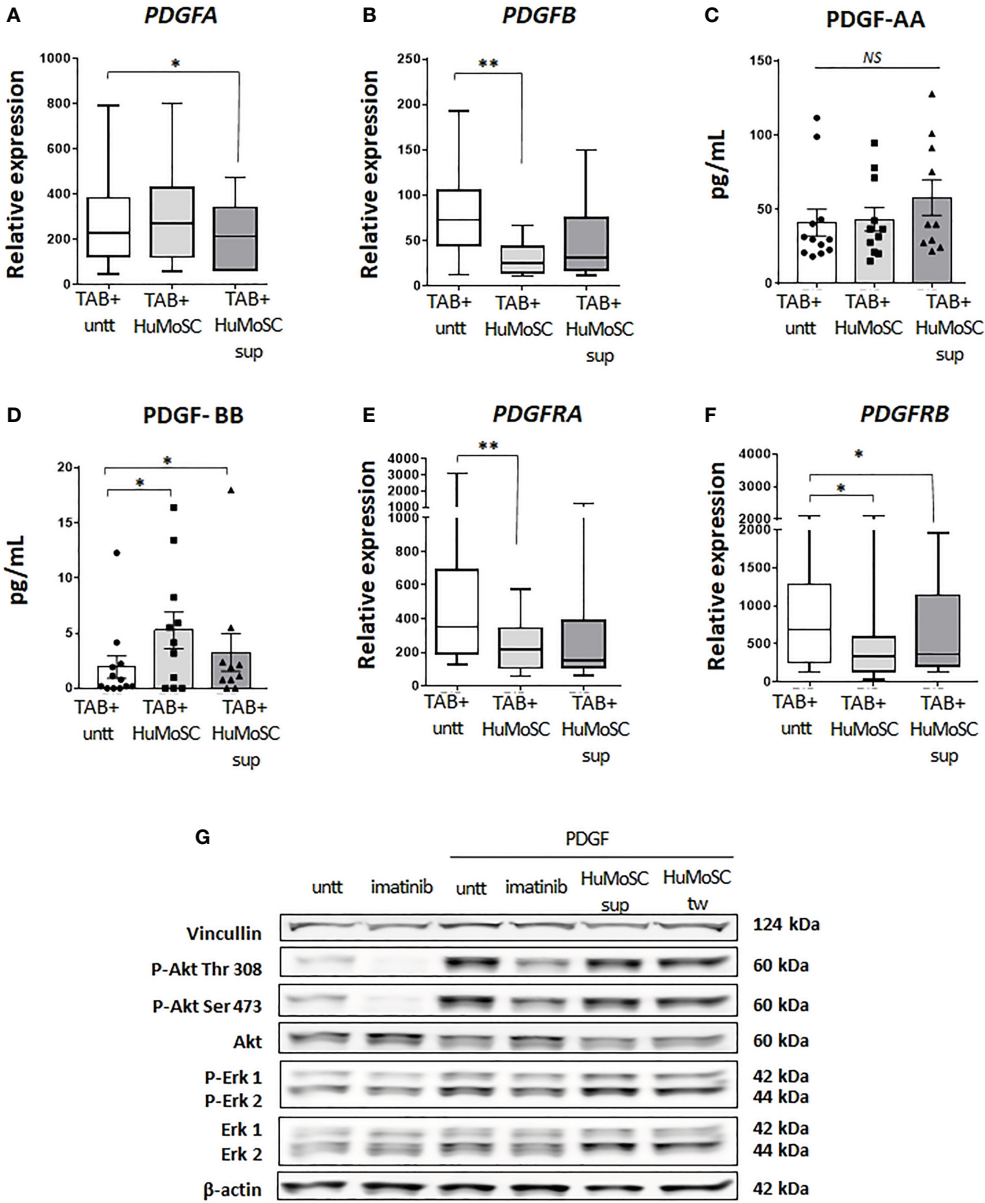
Effect of HuMoSC in the PDGF pathway. **(A-D)** effect of HuMoSC on the mRNA expression and production of PDGF after 5 days of *ex vivo* cultures of temporal arteries affected by GCA (TAB+, n=12) cultivated with or without HuMoSC (250.10^3^/mL) (n=11) or supernatant of HuMoSC (25%) (n=12). mRNA expression in TAB+ **(A, B)** and protein level in the supernatant of culture **(C, D)**. **(E, F)** mRNA expression of PDGFRA **(E)** and PDGFRB **(F)** after 5 days of *ex vivo* cultures of temporal arteries affected by GCA (TAB+, n=12) cultivated with or without HuMoSC (250.10^3^/mL) (n=11) or supernatant of HuMoSC (25%) (n=12). **(G)** study of the phosphorylation of Akt and Erk by western blot in primary cultures of temporal artery derived VSMC. VSMC were pre-treated with imatinib (1 µM, positive control for inhibition of phosphorylation), HuMoSC supernatant (25%) or HuMoSC (250.10^3^/mL separated by a transwell [tw]) during 1 hour and then cultivated alone or in the presence of PDGF-AB (20 ng/ml) for 30 minutes. VSMC were collected in 125 µL of RIPA buffer before performing western blots. β-actin and vinculin were stained as standards (representative result of 2 independent experiments). Whisker boxes show the median (horizontal bar), the IQR (limits of the box) and extreme values (error bars). Histograms show the median (horizontal bar) and the IQR (error bars). *P* is the result of Wilcoxon tests. Significant results are shown (*P < 0.05, **P < 0.01, NS, Not Significant) otherwise non-significant.

Given these data, we evaluated the activity of HuMoSCs in the PDGF pathway. In the model of *ex vivo* cultures of temporal arteries, we observed a mild decrease in the level of *PDGFA* expression in the presence of HuMoSC supernatant (**Figure 4A**), which was not confirmed at a protein level (**Figure 4C**). Additionally, HuMoSCs decreased more significantly the mRNA expression of *PDGFB* (**Figure 4B**), but paradoxically PDGF-BB concentration increased in the presence of HuMoSCs or their supernatant (**Figure 4D**). Taken together, these results do not support a major effect of HuMoSCs or their supernatant on PDGF-AB production.

PDGF are expressed as dimers (AA, AB and BB) that bind to their receptors, which are also composed of two chains: PDGF-Rαα and PDGF-Rαβ bind all types of PDGF dimers whereas PDGFR-ββ only links PDGF-BB. Then, it activates several intracellular pathways inducing the phosphorylation of Akt and Erk and ultimately the activation of the mTOR pathway, resulting in cellular proliferation (17).

The study of *ex vivo* cultures of TAB revealed that treatment with HuMoSCs, and to a lesser extent HuMoSC supernatant, resulted in a significant decrease in the level of expression of both PDGF receptor chains (**Figures 4E, F**).

Further experiments revealed that HuMoSCs or their supernatant had no immediate effect on the phosphorylation of Erk1, Erk2 and Akt (Thr 308 and Ser 473) (**Figure 4G**).

Taken together, these results suggest that HuMoSCs, or their supernatant, may act *via* a decrease in PDGF receptor expression more than through a rapid decrease in activation of the PDGF signaling pathway.

### HuMoSCs act on VSMCs through the inhibition of mTOR activity

**Figure 5:**
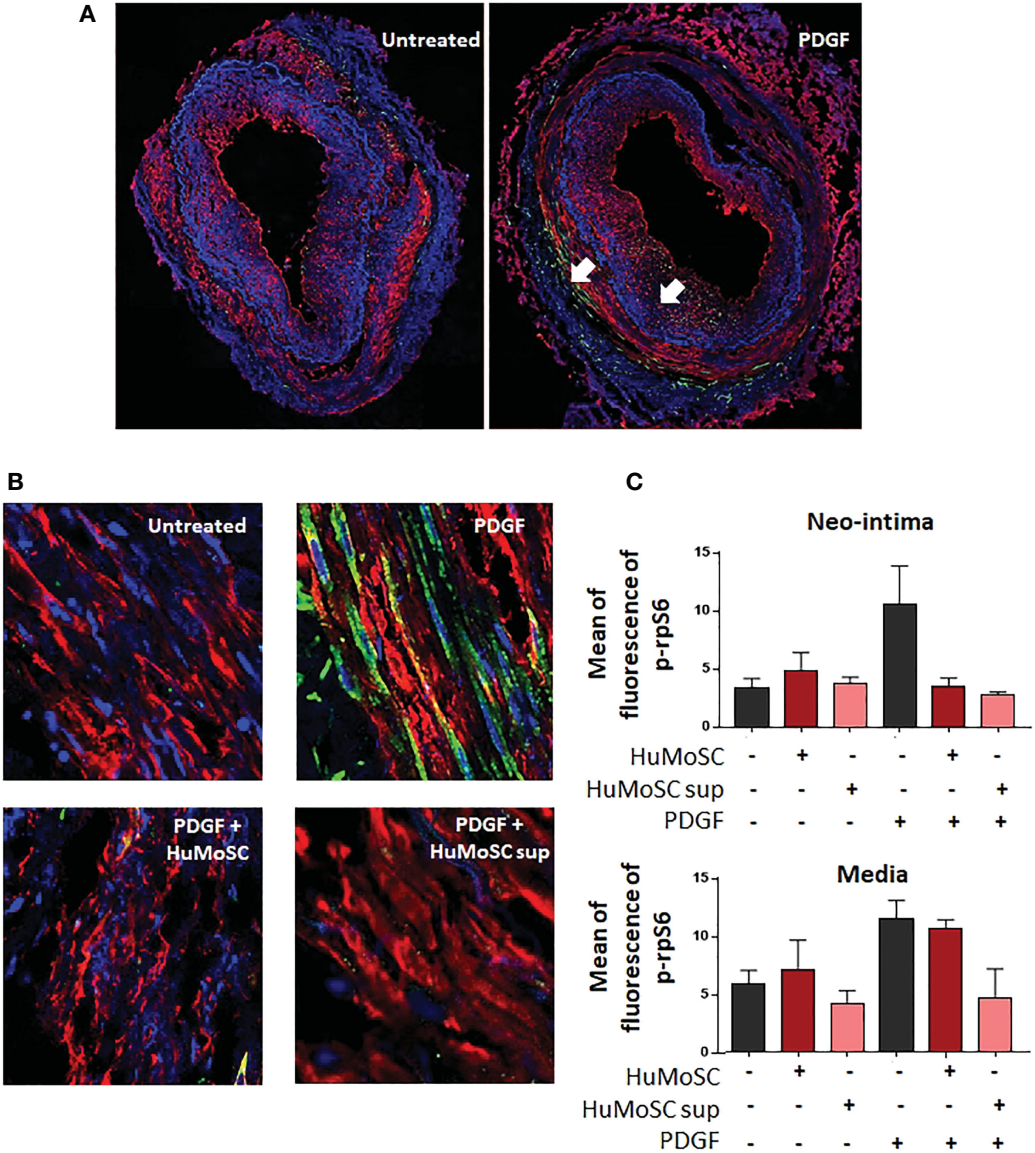
Effect of HuMoSC in the mTOR pathway. **(A, B)** Confocal microscopy analysis of temporal arteries affected by GCA cultivated during 5 days alone or in the presence of PDGF (20 ng/mL), HuMoSC (250.10^3^/mL) or supernatant of HuMoSC (25%). Pictures show staining of α-SMA (red), p-rpS6 (green) and nuclei (DAPI, blue). p-rpS6, whose expression is shown by the white arrows, is a metabolite of mTOR. Thus, the intensity of the green staining reflects the activation of the mTOR complex. Pictures show the entire artery **(A)** and magnifications (x 40) in the media **(B)**. **(C)** Mean ± SEM fluorescence intensity of p-rpS6 staining normalized to background noise was calculated in the neo-intima and the media of each temporal artery using ImageJ fiji software (untreated TAB+ [n=5], HuMoSC [n=3], HuMoSC supernatant [n=3], PDGF [n=4], PDGF + HuMoSC [n=2], PDGF + HuMoSC supernatant [n=2]).

mTOR acts by activating the p70 ribosomal S6 kinases: S6K1 and S6K2. S6K1 phosphorylates the S6 protein on the 40S ribosomal subunit and leads to cell proliferation. Therefore, we evaluated mTOR activity by measuring the mean fluorescence intensity of phospho-S6 ribosomal protein in cultured arteries. We observed that PDGF induced activation of the mTOR pathway in the media and the neointima (**Figure 5A**), and that was strongly decreased in the presence of HuMoSCs or HuMoSC supernatant (**Figure 5B**). Interestingly, this effect seemed stronger in the neointima than in the media (**Figure 5C**). By contrast, mTOR was poorly activated in the adventitia (**Supplementary Figure 1**).

### HuMoSCs migrate to the arterial wall through interaction between CCR5 and its ligands

**Figure 6:**
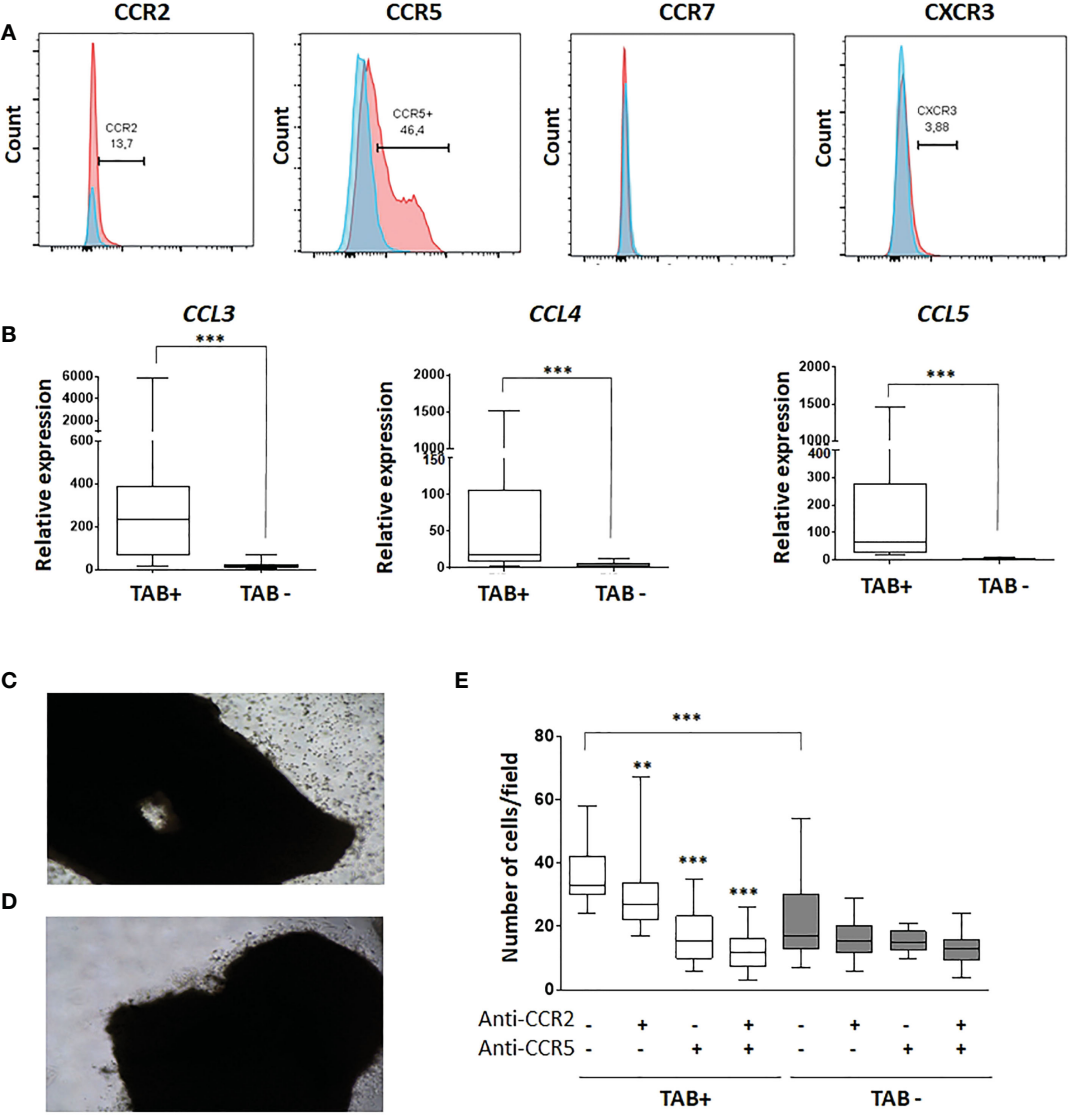
Recruitment of HuMoSC in GCA arteries implicates an interaction between CCR5 and its ligands. **(A)** representative flow cytometry analysis of HuMoSC. Blue histogram is the isotype control and red histograms are staining of HuMoSC after CD14 gating (representative of 10 independent experiments). **(B)** mRNA expressions of *CCL3*, *CCL4* and *CCL5* in positive TAB (n=12) and negative TAB (n=9) after 5 days of *ex vivo* culture in MATRIGEL^®^. **(C, D)** Picture of a representative positive TAB after 4 hours of culture in MATRIGEL with **(C)** or without **(D)** HuMoSC. **(E)** Migration assay in Boyden chamber. The lower chamber was filled with supernatants of negative or positive TABs cultivated in MATRIGEL during 5 days. A total of 30.10^3^ HuMoSC were added in the upper chamber with or without antagonist of CCR2 (calbiochem, 100 nM) and/or CCR5 (maraviroc, 1 µM). After 4.5-hour incubation, the polycarbonate membrane was stained with hematoxylin and eosin and washed before counting number of cells/field. Each condition was performed in quadruplet and 2 fields were analyzed per condition. The experiment was repeated 3 times independently. For statistical analyses, each condition was compared with the condition in which there was no treatment with antagonists of CCR2 and CCR5, except otherwise specified. Statistics: Whisker boxes show median (horizontal bar), IQR (limits of the box) and extreme values (error bars). *P* is the result of Mann Whitney test (untreated TAB+ *vs.* untreated TAB-) or Wilcoxon tests (untreated TAB+ *vs*. TAB+ treated with anti-CCR2, anti-CCR5 or both): **P < 0.01; ***P < 0.001.

We then explored how HuMoSCs might be recruited to GCA lesions to support the cells’ therapeutic potential. Flow cytometry analysis of HuMoSCs showed that they predominantly express CCR5 and to a lesser extent CCR2, very few CXCR3 and no CCR7 (**Figure 6A**). In addition, mRNA levels of chemokines that link CCR5 (i.e. CCL3, CCL4 and CCL5) were strongly expressed in GCA-TAB but not in healthy TAB (**Figure 6B**). Then, we demonstrated that this interaction between CCR5 and its ligands explains the rapid recruitment of HuMoSCs within the arterial wall visualized when HuMoSCs are added in TAB cultured *ex vivo* in MATRIGEL (**Figures 6C, D**).We therefore performed Boyden chamber migration assays in the presence of CCR2 and/or CCR5 antagonists, which showed that HuMoSCs migrate more toward the lower chamber when it contains supernatant from GCA-TAB, whereas they hardly migrate toward supernatant from healthy TAB (P<0.001). Migration decreased significantly in the presence of a CCR2 antagonist (P=0.005) and even more so in cases of CCR5 blockade (P<0.001), suggesting that HuMoSCs are able to be recruited to the arterial wall through an interaction between CCR5 and its ligands, and to a lesser extent between CCR2 and its ligands, which are all produced within arteries affected by GCA (**Figure 6E**).

## Discussion

Recent advances in the understanding of GCA pathogenesis have led to the identification of new therapeutic targets, such as IL-6, IL-17, GM-CSF or JAK/STAT signaling proteins (18). This has led to the development of new treatments with proven (12, 13, 19, 20) or possible (21–25) efficacy in GCA. However, most of these treatments appear to be suspensive or ineffective in all patients. For example, 15–26% of patients experience GCA relapses during treatment with tocilizumab and about half relapse within 6 months of stopping it (13, 26, 27). Moreover, most of these treatments act on inflammation but have not been explored on vascular remodeling, which causes the most severe complications and highlights that there are still unmet needs in GCA (28).

The use of immunosuppressive cells, such as mesenchymal stem cells (MSCs), has already been proposed in systemic sclerosis. MSCs are characterized by immunosuppressive, antifibrotic and proangiogenic capabilities and may be a promising alternative option for the treatment of systemic sclerosis (29). Our team has developed an original cell therapy of myeloid origin, HuMoSCs, which are generated by inflammatory stimuli and have strong immunosuppressive properties. Previous works have shown that, *in vitro*, HuMoSCs suppress T-cell proliferation and activation and are also able to expand Treg (15). Furthermore, we demonstrated that the immunosuppressive functions of HuMoSCs are preserved even in an inflammatory environment or in the presence of immunosuppressive treatments, which is an advantage for the treatment of autoimmune diseases (30).

The *ex vivo* culture model of temporal arteries in MATRIGEL which we used in this study allows us to study the mechanisms of vascular remodeling rather than vascular inflammation (3). Despite this model’s limitations, our results suggest that HuMoSCs or their supernatant decrease the expression of genes involved in the recruitment of monocytes (*CCL2*) and T-cells (*CXCR3*) to the arterial wall (31). The decrease in *CCR2* expression also suggests that arterial monocytes may change their phenotype to a less proinflammatory one (32).

In addition to these data suggesting an effect of HuMoSCs on vascular inflammation, our results were even clearer with respect to their effect on vascular remodeling since HuMoSCs or their supernatant decreased the expression of genes and/or the production of most extracellular matrix proteins (collagen 1, collagen 3, fibronectin) and mediators involved in neoangiogenesis (VEGF). CCL2, which is known to be produced by vessel wall components, such as VSMCs and endothelial cells, contributes to the inflammatory state in GCA but also stimulates angiogenesis through CCR2 on endothelial cells (33). Their expressions were downregulated in the presence of HuMoSCs. Together these data suggest an anti-remodeling effect of HuMoSCs, which was confirmed by *in vitro* experiments that clearly showed that HuMoSC supernatant inhibits VSMC migration and proliferation when stimulated by PDGF, as is the case during GCA (6).

Investigation of the mechanisms involved in this anti-vascular remodeling effect suggests that it is unrelated to modulation of PDGF production but rather to a decrease in PDGF receptor expression. In addition, we found that HuMoSCs (or their supernatant) clearly induced a decrease in mTOR complex activity in cultured arteries, but without modifying rapid phosphorylation level of Erk and AKT in primary cultures of VSMC exposed to PDGF, even though these pathways are involved in intracellular PDGF signaling and are canonical for mTOR activation (17). These results may seem contradictory. However, it can be explained in several ways. Firstly, the timing of the analysis, since AKT and Erk phosphorylation were measured in VSMC cultured *in vitro* after 1 hour of treatment, whereas PDGF receptor expression and mTOR activity were studied in arteries cultured in MATRIGEL for 5 days. Therefore, under the conditions of our experiments, rapid changes in ERK or Akt phosphorylation in isolated VSMC were not apparent, but we cannot rule out an effect at later time points. The other plausible explanation, not inconsistent with the first, is that HuMoSCs (or their supernatant) could inhibit the mTOR complex by acting on another PDGF-activated pathway, such as AMP-activated protein kinase (AMPK). Indeed, telmisartan has been shown to inhibit rat VSMC proliferation *via* AMPK-dependent inhibition of mTOR phosphorylation (34). Along this line, another study demonstrated that jujuboside B antagonizes PDGF-BB-induced phenotypic switch, proliferation and migration of VSMCs partly through AMPK/PPAR-γ pathway (35).

Our results also provide new insights into the mechanisms of action of HuMoSCs by showing that they are able to migrate to the site of the inflammatory response *via* an interaction between CCR5 and its ligands. Furthermore, the fact that HuMoSCs inhibit the mTOR complex is therapeutically relevant since previous data have shown that the mTORC1 pathway plays a central role in driving T-cell inflammation and vascular injury in large vessel vasculitis (36, 37).

This proof-of-concept study highlights the cells’ therapeutic potential in GCA. Indeed, autologous HuMoSCs, which can be generated from GCA patients (unpublished data), have immunosuppressive properties to inhibit T-cell proliferation and their polarization into Th1 and Th17 cells, and in this study we also showed that these immunosuppressive cells are able to decrease vascular remodeling, which is an unmet need in GCA treatment. Together, our results show that HuMoSCs, or their supernatant, could represent an autologous therapy in GCA patients. In the conditions of our experiments HuMoSCs did not impact IL-1β and IL-6 production, although we cannot exclude an effect at later time points. Therefore, HuMoSCs or their supernatant smight be used in addition to other GCA treatments to target pathways that are currently poorly controlled by current therapies, such as those involved in vascular remodeling.

Finally, our results also suggest that such therapy could be promising in diseases in which the fibrotic remodeling process plays a major role and for which fewer effective treatments are available, such as systemic sclerosis.

## Conclusion

In our GCA model, HuMoSCs triggered a decrease in markers of vascular inflammation and especially vascular remodeling. The mechanism depends, at least partially, on a decrease in the expression of PDGF receptors and inhibition of the mTOR complex. This suggests that these cells, or their supernatant, have a potential interest in GCA treatment and could represent an alternative therapeutic strategy to reduce the doses of immunosuppressive drugs and GCs and to better control vascular remodeling. Our work also opens new perspectives for the treatment of diseases involving tissue remodeling such as fibrosis, e.g. systemic sclerosis.

## Data availability statement

The raw data supporting the conclusions of this article will be made available by the authors, without undue reservation. 

## Ethics statement

The patients whose TABs were studied herein gave written informed consent in accordance with the Declaration of Helsinki (Collection DC-2016-2675). The patients/participants provided their written informed consent to participate in this study.

## Author contributions

MS, BB, and MCC were the principal investigators and take primary responsibility for the paper. MS, HG, GE-F, P-HG, CC-G, SA, BB, and MCC recruited the patients. MS, CoG, MC-B, HG, ClG, CC, RA-R, MC, GT, and LM performed the laboratory work. MS and CoG did the statistical analyses. MS, BB, and MCC coordinated the research. MS, CoG, MCC, and BB contributed to data interpretation. MS, CoG, PS, BB, and MCC drafted the manuscript. All authors contributed to the article and approved the submitted version. 
