# Supplementary material for: Human monocyte-derived suppressive cells (HuMoSC) for cell therapy in giant cell arteritis
Source: Front Immunol. 2023 Feb 21;14:1137794. doi: 10.3389/fimmu.2023.1137794 (PMC9989212; doi:10.3389/fimmu.2023.1137794)
Supplement: Supplementary file 1 [file DataSheet_1.docx]

**SUPPLEMENTARY TABLES**

**Sup Table 1.** Main characteristics of GCA patients at diagnosis (n=12).

| ***Age (years), median [range]*** | 84 [83-92] |
| --- | --- |
| ***Sex ratio (M/F)*** | 7/5 |
| ***Clinical characteristics at diagnosis, n (%)***  *Cranial symptoms*  Headache  Jaw claudication  Vision loss  Permanent *  Transient  Diplopia  Temporal artery abnormality  *Systemic symptoms*  Fever  Weight loss  *Polymyalgia rheumatica* | 10 (83)  5 (42)  5 (42)  3 (25)  2 (17)  3 (25)  7 (58)  0 (0)  7 (58)  4 (33) |
| ***Positive temporal artery biopsy, n (%)***  ***GC started at the time of TAB, n (%)***  ***Median (IQR) delay (days) of GC treatment before TAB*** | 12 (100)  8 (67)  2 (0.25-4.75) |
| ***Laboratory findings at diagnosis, median (IQR)***  ESR (mm/hr)  CRP (mg/L)  Hemoglobin (g/dL)  Platelet count (G/L) | 80 (46-112)  26 (21-133)  11.7 (10.2-13.4)  389 (271-465) |

CRP: C-reactive protein; ESR: erythrocyte sedimentation rate; GC: glucocorticoids

*all permanent vision losses were related to acute ischemic anterior neuropathy

**Sup Table 2**: List of TaqMan probes.

|  | **Gene** | | **Probe reference** | |  |
| --- | --- | --- | --- | --- | --- |
| ***Chemokines*** |  | |  | |  |
| CCL2  CCL3  CCL4  CCL5 | *CCL2*  *CCL3*  *CCL4*  *CCL5* | | Hs00234140_m1  Hs00234142_m1  Hs99999148_m1  Hs00174575_m1 | |  |
| ***Chemokine receptors*** | | |  | |  |
| CCR2 | *CCR2* | | Hs01560352_m1 | |  |
| CXCR3 | *CXCR3* | | Hs00171041_m1 | |  |
|  |  | |  | |  |
| ***Cell activation*** |  | |  | |  |
| HLA-DR chain α | HLADRA | | Hs00219575_m1 | |  |
|  |  | |  | |  |
| ***Cytokines*** | | |  | |  |
| IL-1β | *IL1B* | Hs01555413_m1 | |  |  |
| IL-6 | *IL6* | Hs00985639_m1 | |  |  |
| ***Growth factors*** | | |  | |  |
| PDGF-A | *PDGFA* | | Hs00234994_m1 | |  |
| PDGF-B | *PDGFB* | | Hs00234042_m1 | |  |
| VEGF | *VEGFA* | | Hs00900055_m1 | |  |
| ***Receptors of growth factors***  PDGF-RA | *PDGFRA* | | Hs00998018_m1 | |  |
| PDGF-RB | *PDGFRB* | | Hs01019589_m1 | |  |
| ***Matrix proteins*** | | |  | |  |
| Collagen I A1 | *COL1A1* | | Hs00164004_m1 | |  |
| Collagen III A1 | *COL3A1* | | Hs00164103_m1 | |  |
| Fibronectine | *FN1* | | Hs01549976_m1 | |  |
|  |  | |  | |  |
| ***Others*** |  | |  | |  |
| β-glucuronidase | *GUSB* | | Hs99999908_m1 | |  |

**Sup Table 3**: List of antibodies for flow cytometry, confocal microscopy and western blot

| **Antibodies** | **Source** | **Application** |
| --- | --- | --- |
| Anti-human CCR7-PE | eBioscience | Flow cytometry |
| Anti-human CXCR3-PECy7 | eBioscience | Flow cytometry |
| Anti-human CCR5-APC | BD bioscience | Flow cytometry |
| Anti-human CCR2-Alexa Fluor 488 | R&D systems | Flow cytometry |
| Anti-human CD14-APC | eBioscience | Flow cytometry |
| Anti-human CD33-FITC | eBioscience | Flow cytometry |
| Anti-human CD3-BV510 | biolegend | Flow cytometry |
| Anti-human CD4-PECy7 | eBioscience | Flow cytometry |
| Anti-human IL-17-PE | eBioscience | Flow cytometry |
| Anti-human IFN-γ-APC | eBioscience | Flow cytometry |
| Rabbit anti-human αSMA | Abcam | Immunofluorescence and western blot |
| Mouse anti-human αSMA | Abcam | Immunofluorescence and western blot |
| Donkey anti mouse A555 (red) | Life technologies | Immunofluorescence |
| Donkey anti goat A555 (red) | Life technologies | Immunofluorescence |
| Donkey anti mouse A647 (far red) | Life technologies | Immunofluorescence |
| Donkey anti rabbit A488 (green) | Life technologies | Immunofluorescence |
| Rabbit anti-human phospho-S6 ribosomal protein (Ser 235/236) | Cell signaling | Immunofluorescence |
| Rabbit anti-vincullin | Abcam | Western blot |
| Mouse anti-β-actin | Sigma Aldrich | Western blot |
| Rabbit anti-phospho AKT (Thr 308) | Cell signaling | Western blot |
| Rabbit anti-phospho AKT (Ser 473) | Cell signaling | Western blot |
| Rabbit anti-AKT | Cell signaling | Western blot |
| Rabbit anti-phospho p44/p42 MAPK (Erk1/2) | Cell signaling | Western blot |
| Rabbit anti-total MAPK (Erk1/2) | Cell signaling | Western blot |

**SUPPLEMENTARY FIGURES**

**
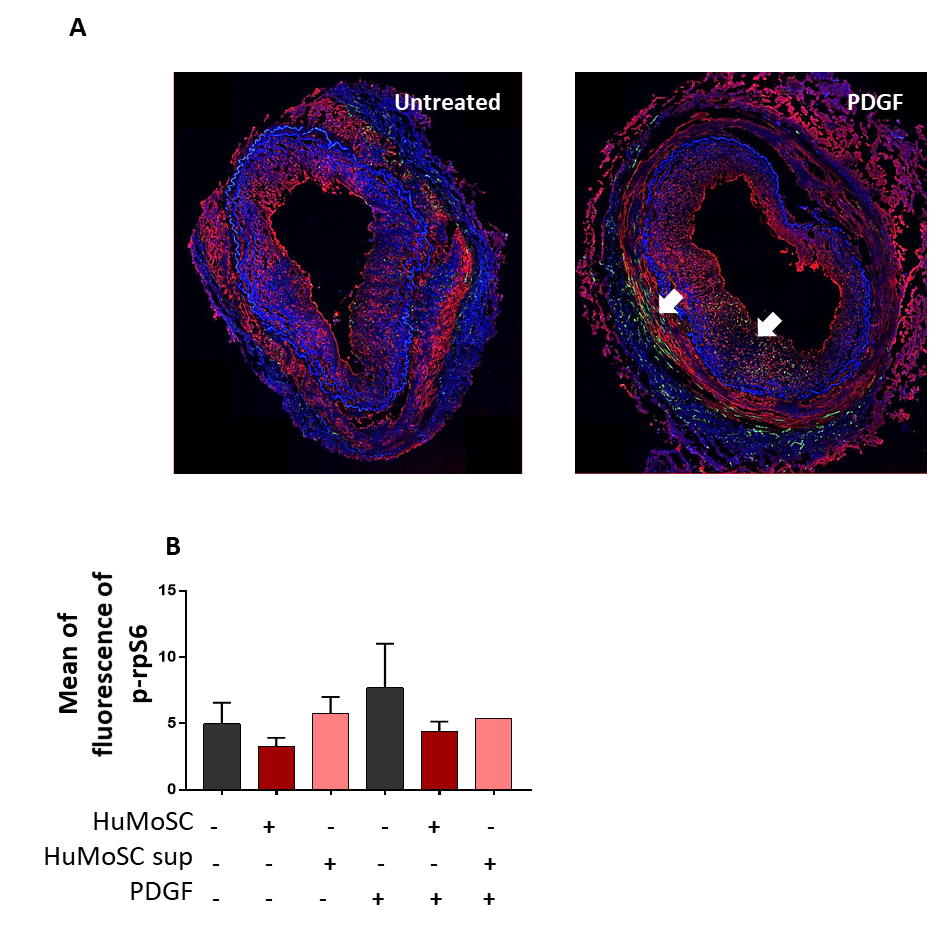
**

**Figure S1: mTOR activity in the adventitia.** Confocal microscopy analysis of temporal arteries affected by GCA cultivated during 5 days alone or in the presence of PDGF (20 ng/mL), HuMoSC (250.10^3^/mL) or HuMoSC supernatant (25%). Mean ± SEM fluorescence intensity of p-rpS6 staining normalized to background noise was calculated in the adventitia of each temporal artery using ImageJ fiji software (untreated TAB+ [n=5], HuMoSC [n=3], supernatant of HuMoSC [n=3], PDGF [n=4], PDGF + HuMoSC [n=2], PDGF + supernatant of HuMoSC [n=2]).
